# FTO-mediated m6A demethylation regulates PGC-1α-dependent mitochondrial biogenesis to attenuate aluminum-induced neuronal senescence

**DOI:** 10.1038/s41598-026-51674-w

**Published:** 2026-05-07

**Authors:** Zhaoya Jin, Shuai Li, Jinzhu Yin, Xiaoyu He, Ru Wang, Yang Liu, Huifang Zhang

**Affiliations:** 1 Department of Occupational Health, School of Public Health, Shanxi Medical University, Taiyuan, China; 2 Key Laboratory of Environmental Health Impairment and Prevention in Shanxi, Taiyuan, China; 3 MOE Key Laboratory of Coal Environmental Pathogenicity and Prevention in Shanxi, Taiyuan, China; 4 First Clinical Medical College, Shanxi Medical University, Taiyuan, China; 5 Department of Neurosurgery, Datong Key Laboratory of Nervous systems Disease Prevention and Treatment for Coal Mine workers, Shanxi Health Commission Key Laboratory of Nervous System Disease Prevention and Treatment, Sinopharm Tongmei General Hospital, Datong, 037003 Shanxi China

**Keywords:** Aluminum, FTO, m6A methylation, PGC-1α, Mitochondrial biogenesis, Senescence, Cell biology, Genetics, Molecular biology, Neuroscience

## Abstract

Aluminum (Al) exposure is increasingly recognized as a risk factor for neurodegenerative disorders; however, the epitranscriptomic mechanisms linking environmental Al toxicity to neuronal senescence and mitochondrial alterations remain poorly understood. We hypothesized that impairs mitochondrial biogenesis and may disrupt mitochondrial homeostasis through dysregulation of N6-methyladenosine (m6A) RNA modification mediated by the m6A demethylase fat mass and obesity-associated protein (FTO). HT22 mouse hippocampal neurons were exposed to aluminum maltolate (60–240 µmol/L). An FTO-overexpression model was established. MeRIP-seq and RNA-seq were performed to assess m6A and transcriptomic changes. Mitochondrial status, particularly mitochondrial biogenesis–related indicators, was evaluated via ATP and mtDNA levels. SA-β-Gal staining assessed senescence. The expression of senescence-associated markers (e.g., p16, p21, HMGA1) and mitochondrial regulatory factors (e.g., FTO, PGC-1α, NRF-1, NRF-2, TFAM) was examined by qPCR and Western blotting. Aluminum exposure globally increased m6A methylation (7,068 peaks upregulated), affecting pathways linked to senescence and neurodegeneration. PGC-1α showed increased m6A and decreased expression. Aging markers (P16, P21, HMGA1) were upregulated, while mitochondrial biogenesis–related indicators (ATP levels, mtDNA copy number, and the PGC-1α axis) were reduced, indicating impaired mitochondrial biogenesis. FTO expression was suppressed by aluminum but overexpression of FTO reversed these effects, reducing m6A levels, restoring PGC-1α expression, partially restoring mitochondrial biogenesis-related parameters, and attenuating senescence. Our findings suggest that aluminum induces neuronal senescence by inhibiting FTO, increasing m6A methylation, and downregulating PGC-1α-mediated mitochondrial biogenesis. The FTO–m6A–PGC-1α axis plays a critical role in aluminum neurotoxicity.

**Supplementary Information:**

The online version contains supplementary material available at 10.1038/s41598-026-51674-w.

## Introduction

 With the increasing prevalence of industrial applications and environmental exposure, the potential health risks of aluminum (Al) have attracted growing attention. Chronic low-dose Al exposure is of particular concern, as it can lead to progressive accumulation in the body and exert neurotoxic effects through multiple molecular pathways^1,2^. Evidence indicates that Al is capable of crossing the blood-brain barrier (BBB), where it triggers oxidative stress, mitochondrial dysfunction or alterations, and calcium homeostasis disruption, ultimately resulting in neuronal apoptosis and synaptic impairment^3^. These neurotoxic effects are implicated in the pathogenesis of several neurodegenerative diseases, including Alzheimer’s disease (AD) and Parkinson’s disease (PD)^4^. Notably, neuropathological changes such as cellular senescence, chronic inflammation, and mitochondrial dysfunction or alterations are frequently observed in the brain even prior to the clinical onset of neurodegeneration^5^. Cellular senescence is a state of permanent cell cycle arrest triggered by intrinsic or extrinsic stressors, characterized by proliferative decline, altered morphology, metabolic reprogramming, and the emergence of a senescence-associated secretory phenotype (SASP)^6^. Neurons can still exhibit senescence-associated features under pathological stimuli, including DNA damage accumulation, heterochromatin formation, elevated SA-β-Gal activity, and increased secretion of reactive oxygen species (ROS) and inflammatory mediators^7^. Jurk et al. reported a significant upregulation of senescence markers such as p16^INK4a and p21 in neurons from aged mice and human postmortem brain tissues, suggesting that neuronal senescence may play a pathological role in neurodegenerative progression^8^.

Mitochondria serve as central regulators of cellular energy metabolism and play a pivotal role in maintaining neuronal viability and function. Mitochondrial damage and impaired biogenesis have been identified as key drivers of cellular senescence^9^. Through oxidative phosphorylation, mitochondria generate ATP to meet the high energy demands of neurons, while simultaneously serving as both major sources and targets of ROS^10^. Disruption of mitochondrial biogenesis leads to decreased ATP production, ROS accumulation, mtDNA mutations, and activation of pro-inflammatory pathways, all of which contribute to cellular dysfunction and aging^11^. Mitochondrial biogenesis is essential for maintaining both mitochondrial quantity and quality. This process is orchestrated by a network of transcriptional regulators, among which peroxisome proliferator-activated receptor gamma coactivator-1 alpha (PGC-1α) is considered a master regulator. PGC-1α activates nuclear respiratory factors 1 and 2 (NRF-1/2), which in turn induce mitochondrial transcription factor A (TFAM), promoting mtDNA replication and transcription of mitochondrial genes^12^. Studies have shown that PGC-1α expression is significantly reduced in aged tissues and models of neurodegenerative diseases. Loss of PGC-1α impairs brain structure and cognitive function, whereas its overexpression improves mitochondrial function and attenuates neurodegeneration^13,14^.

Recent advances in epigenetic regulation have highlighted the importance of post-transcriptional modifications, particularly N6-methyladenosine (m6A) methylation of mRNA, in neural development, synaptic plasticity, metabolic regulation, and aging. m6A is the most prevalent internal modification in eukaryotic mRNA and is dynamically regulated by methyltransferase complexes (METTL3, METTL14, WTAP) and demethylases (FTO, ALKBH5)^15,16^. Fat mass and obesity-associated protein (FTO), the first identified m6A demethylase, is highly expressed in the adult central nervous system and regulates neural stem cell proliferation, neurotransmitter release, and learning and memory^17^. Deficiency of FTO leads to excessive m6A accumulation, disrupted neuronal transcriptomic programs, impaired mitochondrial function, and cognitive deficits^18^. Emerging evidence suggests that FTO may modulate mitochondrial morphology and biogenesis by regulating m6A methylation of mitochondrial-related genes^19^. Although previous studies have investigated Al-induced neurotoxicity, mitochondrial dysfunction, neuronal senescence, and m6A epitranscriptomics separately, the potential crosstalk between these pathways remains poorly understood. Specifically, it is unclear whether FTO-mediated m6A demethylation modulates PGC-1α–dependent mitochondrial biogenesis during Al-induced neuronal aging.

In this study, we hypothesized that Al-induced FTO downregulation impairs mitochondrial biogenesis and may contribute to mitochondrial functional alterations through an m6A-dependent mechanism. To test this hypothesis, we utilized an Al-exposed HT22 cell model as a representative neuronal system. While we recognize the limitations of using a simplified cell line to model complex human neurodegeneration, this approach allows for the precise integration of RNA-seq with MeRIP-seq to characterize the regulatory role of the FTO/PGC-1α axis. By combining molecular profiling with functional assays, this study seeks to elucidate a novel epitranscriptomic mechanism linking environmental Al exposure to neuronal aging, thereby providing potential targets for the intervention of Al-induced neurotoxicity.

## Materials and methods

### Cell culture

Mouse hippocampal neuronal cell line (HT22) (BeNa Culture Collection, Henan, China) were cultured in DMEM (Gibco, Thermo Fisher Scientific, Waltham, MA, USA) supplemented with 10% fetal bovine serum and 1% penicillin-streptomycin. And the cells were incubated at 37 °C with 5% CO_2_(Gibco, Thermo Fisher Scientific, Waltham, MA, USA). The medium was substituted every two days and were passed at a ratio of 1:3 to 1:5 Upon reaching 80–90% confluence.

### Lentiviral transduction and expression verification

HT22 mouse hippocampal neurons in logarithmic growth phase were seeded in 6-well plates (Corning Incorporated, Corning, NY, USA) at a density of 5 × 10⁴ cells per well and cultured for 24 h to reach approximately 30–40% confluence at the time of infection. Lentiviral transduction was performed using recombinant lentiviruses encoding full-length mouse FTO (pcSLenti-EF1-EGFP-P2A-Puro-CMV-Fto-3xFLAG-WPRE; Cat# H31908, Obio Technology) or the corresponding empty vector (pcSLenti-EF1-EGFP-P2A-Puro-CMV-MCS-3xFLAG-WPRE; Cat# GL180,same vendor) as a negative control. A multiplicity of infection (MOI) of 20 was selected based on preliminary optimization experiments.

The required viral volume was calculated according to the formula: viral volume (µL) = (cell number × MOI/viral titer) ×10. Viral particles were diluted in complete DMEM supplemented with polybrene (5 µg/mL) and added to the cells. After 12–16 h of incubation at 37 °C with 5% CO_2_, the medium was replaced with fresh complete DMEM. To establish a stable cell line and eliminate non-transduced cells, puromycin (2 µg/mL; Sigma-Aldrich) was added to the medium 24 h post-transduction. This antibiotic selection was maintained for 72 h, with the concentration determined by a previously established kill curve for HT22 cells. Cells were maintained for an additional 48–72 h to allow stable transgene expression. Transduction efficiency was evaluated at 72 h post-infection by fluorescence microscopy based on enhanced green fluorescent protein (EGFP) expression. More than 80% of cells exhibited robust green fluorescence, which was further supported by immunofluorescence analysis. The successful overexpression of FTO at protein levels was rigorously verified by Western blot (Fig. 7b). Cells with confirmed FTO overexpression were subsequently subjected to aluminum maltolate treatment and downstream molecular and functional analyses.

### Chemical preparation and treatment

Aluminum maltolate^20^ was prepared by mixing AlCl_3_ and maltol (Sigma-Aldrich, USA) in equal volumes as previously described^20^. The solution was prepared fresh before each use and adjusted to pH 7.4 followed by filtration (0.22 μm). Maltolate was employed as a metal-chelating ligand to enhance the solubility and bioavailability of aluminum at physiological pH. To exclude potential interference from the ligand itself, a maltol-only group (without aluminum) was included as a vehicle control; preliminary experiments confirmed that maltol at the tested concentrations had no significant effect on HT22 cell viability or senescence markers. Based on CCK-8 dose–response assays, three concentrations (60, 120, and 240µM) were selected for subsequent studies. Cells were divided into the following groups: (1) Control (DMEM only); (2) Maltolate control; (3) Al(mal)_3_ treatment (60, 120, 240 µM); (4) OE-NC; (5) OE-NC + Al(mal)_3_; (6) OE-FTO; and (7) OE-FTO + Al(mal)_3_. Following 24 h of treatment, cells were harvested for molecular and functional analyses.

### SA-β-gal staining

Identification of cellular senescence using SA-β-gal assay kit (Beyotime Biotechnology, Shanghai, China). The SA-β-gal staining was performed according to the manufacturer’s protocol and adapted from the method described by Dimri and Dimri (2024)^21^.The same number of HT22 exposed to different treatments in vitro were incubated in 6-well plates. Subsequently, the cells were fixed with 4% formaldehyde(Tianjin Damao Chemical Reagent Co., Ltd., Tianjin, China) for 15 min at room temperature. The cells were washed three times with PBS for 3 min and then incubated with freshly prepared SA-β-gal staining solution overnight at 37 °C in a humidified chamber. The next day, the cells in 6-well plates were washed twice in PBS for 10 min at room temperature. Then, were observed and captured using a microscope equipped with a digital camera (Leica DMi1, Leica Microsystems, Wetzlar, Germany). For each well, at least three fields were captured to calculate the SA-β-gal staining intensity.

### MeRIP-Seq and mRNA sequencing

For RNA-seq and MeRIP-seq analyses, HT22 cells were treated with 120 µM aluminum maltolate for the indicated duration prior to RNA extraction.A total of 25 µg of qualified RNA was dissolved in 50 µL of ultrapure water, followed by the addition of 250 µL of fragmentation buffer. The RNA solution was fragmented at 94 °C for 5 min using a thermocycler. The reaction was then terminated with stop buffer and immediately cooled on ice. Subsequently, 150 µL of pre-equilibrated m6A-Dynabeads was added to the fragmented RNA and incubated at room temperature under rotation (tail-over-head, 7 rpm) for 1 h to selectively enrich m6A-modified RNA. After washing to remove nonspecifically bound materials, the m6A-positive RNA was eluted using pre-heated elution buffer and extracted with acid phenol-chloroform, followed by purification with RNase-free chloroform. The purified RNA was precipitated and resuspended in ultrapure water. RIP libraries were then constructed using 100 ng of RNA (including 100 ng input and 100 ng post-IP fractions) with the Illumina TruSeq Stranded mRNA Library Prep Kit (Illumina, San Diego, CA, USA). Paired-end sequencing (2 × 150 bp) was conducted using the Illumina HiSeq 4000 platform (OE Biotech Co., Ltd., Shanghai, China). The sequencing data generated in this study have been deposited in the GEO database under accession number GSE304247 and GSE304248.

### Data processing

Raw sequencing reads were processed using Trimmomatic software (default parameters: sliding window = 4, average base quality cutoff = 15) to remove adapter-containing reads, poly-N reads, and low-quality reads, resulting in clean data. Ribosomal RNA reads were filtered out using SortMeRNA, and the remaining high-quality reads were mapped to the mouse reference genome (GRCm38/mm10) using HISAT2 with default parameters. The quality of the MeRIP-seq data was assessed using the Guitar R package and deeptools.

MeTDiff software was applied for peak calling to identify m6A-enriched peaks using the corresponding input samples as controls. The peak calling parameters were set as FRAGMENT_LENGTH = 200 and PEAK_CUTOFF_PVALUE = 0.01. Raw P values and adjusted P values controlling for the false discovery rate (FDR) were calculated using the Benjamini–Hochberg method implemented in MeTDiff. Differential m6A peaks were identified based on nominal P values (*P* < 0.05), with FDR-adjusted values provided to control for multiple testing. All identified peaks were annotated according to their genomic features using the ChIPseeker package and visualized with the Integrative Genomics Viewer (IGV).

The expression levels of mRNAs were calculated using the fragments per kilobase of transcript per million mapped reads (FPKM) method. Differentially expressed genes (DEGs) were identified using DESeq2 with the criteria of |log2 FC| > 0.58 and *P* value < 0.05. GO (http://www.geneontology.org) and KEGG (https://www.genome.jp/kegg/) enrichment analyses were subsequently conducted to investigate the biological functions and pathways of the DEGs, with pathways showing *p* < 0.05 considered statistically significant. Heatmaps were generated based on RNA-seq expression data of selected differentially expressed genes and visualized after Z-score normalization.

### RNA extraction and qRT-PCR

Total RNA was extracted using TRIzol (CW Biotech, Beijing, China) according to the manufacturer’s protocol. The A260/A280 ratio was between 1.9–2.1. The RNA was reverse-transcribed using a kit (Takara, Tokyo, Japan) and the fluorescence intensity quantified by RT-PCR using TB Green Premix Ex Taq II (Takara, Tokyo, Japan). The reaction system contained SYBR Premix Ex Taq II (20) (5µL), forward primer (0.4 µL), reverse primer (0.4 µL), ROX reference dye (0.2 µL), cDNA (1.0 µL), and ddh_2_O (3.0 µL). The amplification procedure was: pre-denaturation stage, 95 °C, 30 s; amplification, 40 cycles, 95 °C, 5 s; 60 °C, 34 s; melting curve analysis: 95 °C, 15 s; 60 °C, 1 min; 95 °C, 15 s; The primer sequences (Bioss Biotech Beijing, China) were: FTO FW: 5’-GCAGCTGAATACCCTAAACTG-3’, FTO RV: 5’-AGTCTGGTTCAAGTACTTGT-3’. PGC-1α FW: 5’UUUCUGGGUGGAUUGAAGUGGUGUA-3′, PGC-1α RV: 5’ -ACATGTCCCAAGCCATCCAG-3’. NRF-1 FW: 5’ -GGGAGGTGGATGTAATGTGG-3’, NRF-1 RV: 5’TGGGCCTGGAACTACAACTC-3’. NRF-2 FW: 5’ -AAATTGGGCCACATTACAGGG-3’, NRF-2 RV: 5’-GTTGCATCTCCTGAGAAGCG-3’. TFAM FW: 5’GCGCTCCCCCTTCAGTTTTG-3’, TFAM RV: 5’-GTTTTTGCATCTGGGTTCTGAGC-3’. P16 FW: TTGTGTACCGCTGGGAACG, P16 RV: TTAGCTCTGCTCTTGGGATTGG. P21 FW: GGTGGTGGAGACCTGATGA P21 RV: CGAAGAGACAACGGCACACT. GAPDH FW: 5’-GTGGAGTCATACTGGAACATGTAG-3’, GAPDH RV: 5’-AATGGTGAAGGTCGGTGTG-3’. The 2^−ΔΔCt^ method was used to determine gene expression.

### Determination of ATP content in mouse hippocampal neurons

Detection of cellular ATP using ATP Content Assay Kit (Nanjing Institute of Bioengineering, Jiangshu, China). Cells were collected and resuspended in 300–500 µL of cold double-distilled water, followed by homogenization on ice. A portion of the lysate was taken for protein concentration determination, while the remaining sample was heated in a boiling water bath for 10 min and then mixed thoroughly for 1 min before use. For ATP quantification, blank, standard, sample, and control tubes were prepared. Appropriate volumes of standard solution or sample were added sequentially with substrate solutions I and II and an activator. After thorough mixing, the mixtures were incubated at 37 °C for 30 min. A precipitating reagent was then added, followed by centrifugation at 4000 rpm for 5 min. The supernatant (300 µL) was collected and reacted with a chromogenic reagent for 2 min at room temperature. Subsequently, a stop solution was added, and absorbance was measured at 636 nm. ATP content was calculated using the following formula: ATP (µmol/g protein) = (A_sample_ – A_control_) / (A_standard_ – A_blank_) × C_standard_ × N / Cpr, where C_standard_ is the concentration of the standard (1 × 10^3^ µmol/L), N is the dilution factor, and Cpr is the protein concentration in the sample (g protein/L).

### Determination of mtDNA content in mouse hippocampal neurons

To assess the relative mitochondrial DNA (mtDNA) content (Nanjing Institute of Bioengineering, Jiangshu, China) in HT22, real-time quantitative PCR (RT-qPCR) was performed. This method evaluates mtDNA abundance by comparing the expression levels of the mitochondrial-encoded gene Cyclooxygenase-2 (COX2) with the nuclear-encoded reference gene β-2 microglobulin (B2M). COX2 serves as a mitochondrial marker, while B2M, a stably expressed nuclear gene, is used for normalization. The primer sequences (Bioss Biotech Beijing, China) were as follows: COX_2_ FW: 5′-GCGCACTAATACAAGCACAA-3′, COX_2_ RV: 5′-CAATGGGCAATAGGCATGG-3′, B_2_M FW: 5′-GAACATGTGACTTTGTCACAGCAG-3′, B_2_M RV: 5′-GTATGTGTACCTTCACAGTGGG-3′. PCR reaction conditions were optimized based on previous studies, ensuring primer specificity and stable amplification efficiency. The relative mtDNA content was calculated using ΔCt, where ΔCt = Ct(COX2)- Ct(B2M). The relative abundance of mtDNA was then expressed as 2^–ΔΔCt^, representing the ratio of mitochondrial to nuclear DNA.

### Western blotting

Cells were lysed in RIPA buffer and the total protein quantified with a BCA kit (CW Biotech Beijing, China). Proteins were electrophoresed in SDS and blotted onto a PVDF membrane (EMD Millipore, Bedford, MA, USA). After blocking (5% fat-free milk, room temperature, 2 h), membranes were incubated with primary antibodies (anti-FTO、anti- PGC-1α、anti-NRF-1、anti-NRF-2、anti-TFAM (1:1000, Boster Biological Technology, Wuhan, China) and GAPDH (1:3000, CW Biotech Beijing, China) overnight at 4 °C. Blots were washed (3×TBST) and incubated (2 h, 37 °C) with secondary antibodies (1:3000, CW Biotech). After further washing, an enhanced chemiluminescence chromogenic assay (CW Biotech) was applied to detect the protein content. Band densities were measured using a Chemiluminescence Imaging System (Shanghai Qinxian Scientific Instrument Co., Ltd., China) and analyzed with ImageJ software. Protein expression levels were normalized to GAPDH as the loading control, and GAPDH expression was verified to remain stable under aluminum exposure conditions. Expression was normalized using GADPH. Western blotting procedures followed previously established protocols^22,23^. After electrophoretic transfer, the PVDF membranes were sectioned into several pieces to allow parallel probing of different target proteins. Each membrane section was incubated with the corresponding primary and secondary antibodies. As a result of this procedure, full-length, pre-cut membranes were not retained for some blots. All available original blot images with visible membrane edges, including replicates, have been provided in the Supplementary Information.

### Statistical analysis

All statistical analyses were conducted using GraphPad Prism 10.0 (for graphical representation and basic tests) and SPSS 19.0 (for advanced modeling), with quantitative data expressed as the mean ± SD. Standard deviation (SD) was chosen to describe the dispersion and biological variability of the data among individual samples, which provides a more transparent representation of the experimental measurements than the standard error of the mean (SEM). For two-group comparisons, Student’s t-test was applied after verifying normality (Shapiro-Wilk) and equal variance (Levene’s test). Multi-group analyses employed one-way ANOVA, followed by LSD post-hoc tests for homogeneous variances or Dunnett’s tests for heterogeneous variances (with Brown-Forsythe correction when needed). All statistical inferences adopted a two-tailed threshold of *P* < 0.05.

## Results

### Al exposure alters the m6A methylation profiles in mouse hippocampal neurons

**Fig. 1:**
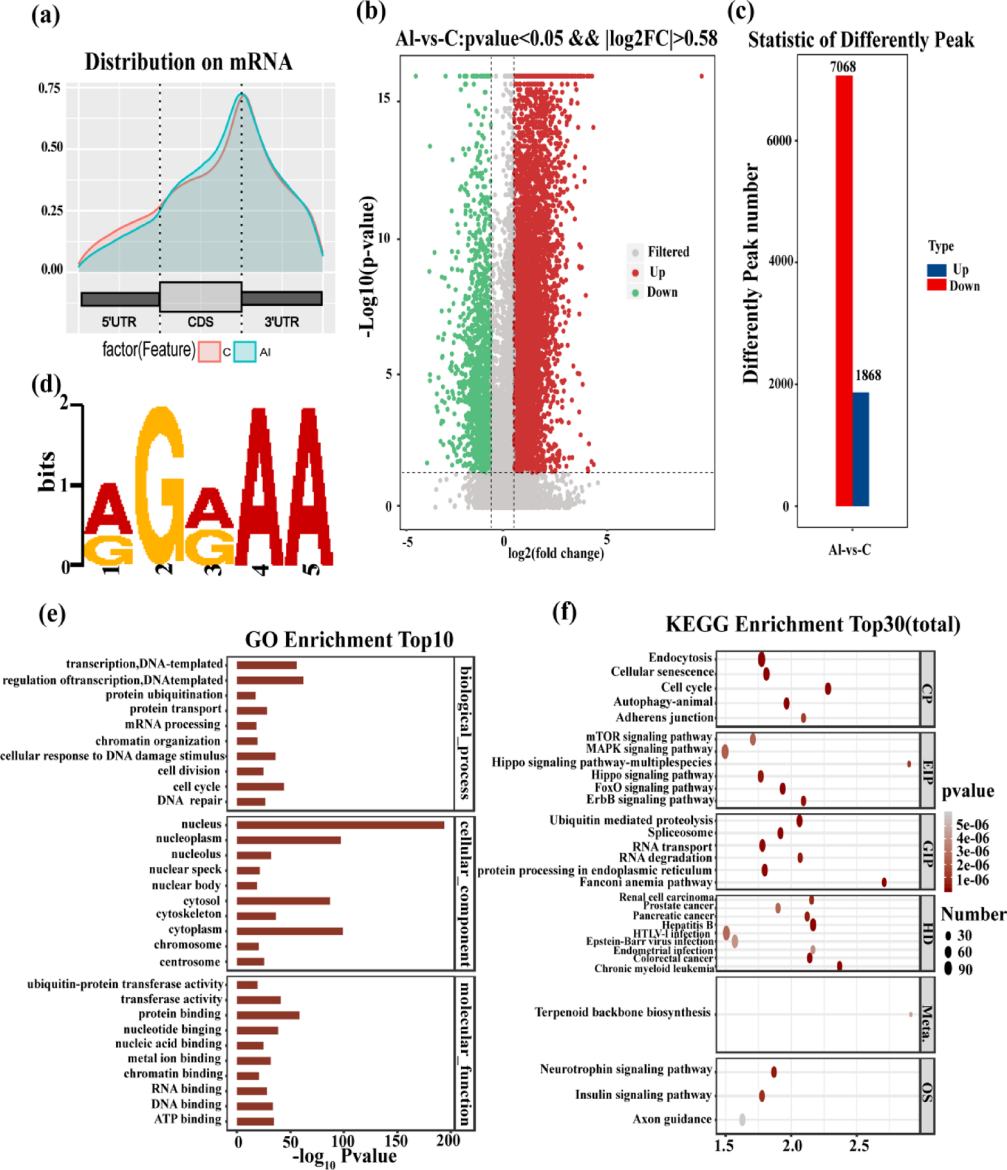
Effect of aluminum exposure on m6A methylation in HT22 cells. (**a**) Meta gene profile showing the distribution of m6A peaks across 5’UTR, CDS, and3’UTR regions. (**b**) Volcano plot illustrating differentially methylated peaks (DMPs)between control and Al treated c ells (cutoff: |log2FC| > 0.58 , *P* < 0.05). (**c**) Bar chartsummarizing the number of hypermethylated and hypomethylated m6A peaks. (**d**) Sequence motif (RGAAR) identified by DREM E analysis, indicating conservedm6A recognition elements. (**e**) GO enrichment analysis of genes containing DMPs (*P* <0.05). (**f**) KEGG pathway enrichment of DMP associated transcripts. Bubble sizerepresents gene count; color scale indicates −log10(P value). Dat a are based on MeRIP seq of *n* = 3 biological replicates per group.

To investigate m6A methylation dynamics following Al exposure, we conducted MeRIP-seq on HT22 cells, which exhibited a globally elevated methylation level upon Al treatment. The analysis identified methylation sites, abundance, and genomic distribution across triplicate biological replicates (12 libraries: 6 input + 6 MeRIP). High-quality data (12.82–15.73 Gb/sample; Q30: 93.67–94.57%; GC: 55.73%) with 75.44–92.04% genome alignment rates (Supplementary Table S1) revealed CDS- enriched m6A peaks (Fig. 1a), suggesting Al-mediated transcriptional/ translational regulation. Motif analysis confirmed the canonical RGAAR (R = A/G) sequence (Fig. 1d), validating peak reliability. Comparative analysis identified 18,551 differential methylated peaks (DMPs) in control samples versus 19,959 DMPs in Al- treated HT22 cells (Supplementary Table S2), demonstrating a global increase in methylation levels following Al exposure. Of these, 8,936 peaks showed significant Al- induced alterations, comprising 7,068 upregulated and 1,868 downregulated sites (*P* < 0.05, fold change > 1.5). Functional annotation (GO/KEGG) highlighted enrichment in DNA-templated transcription, nucleoplasm, and cytosol (GO), alongside pathways like exocytosis, cellular senescence, and neurodegenerative diseases (KEGG) (Fig. 1e, f).

### Differentially expressed genes affected by Al in mouse hippocampal neurons and their functional identification

**Fig. 2:**
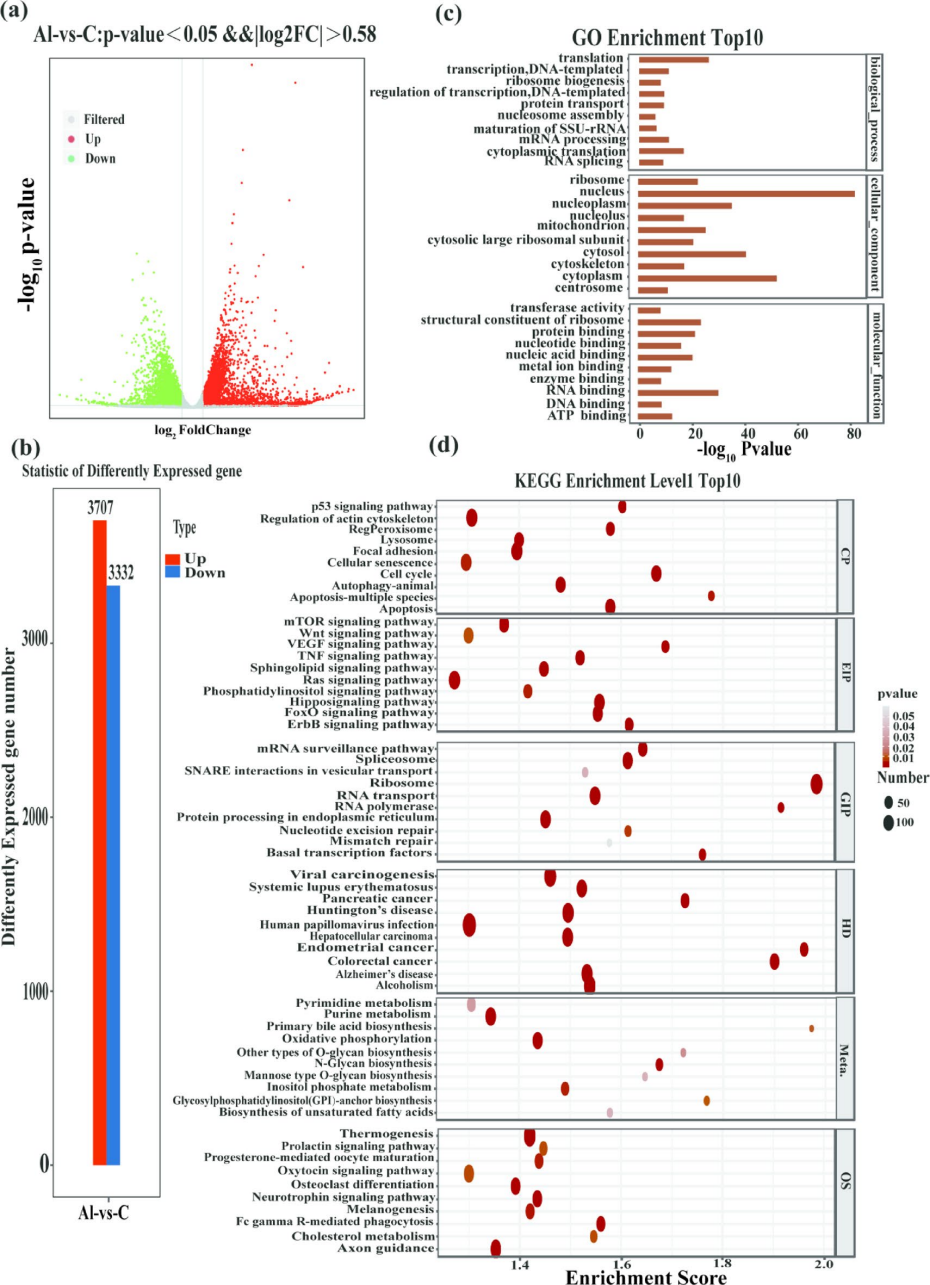
Transcriptomic alterations induced by aluminum exposure in HT22 neurons. (**a**) Volcano plot of differentially expressed genes (DEGs) between control and Al-treated groups (cutoff: |log2FC| > 0.58, *P* < 0.05). (**b**) Counts of upregulated and downregulated DEGs. (**c**) GO enrichment analysis of DEGs at three levels—biological process, cellular component, and molecular function. (**d**) KEGG pathways significantly enriched by DEGs (*P* < 0.05). RNA-seq includes *n* = 3 replicates per group.

As shown in Fig. 2a, to elucidate the molecular mechanisms underlying Al- induced neurotoxicity, we conducted RNA sequencing in HT22 cells, identifying 6,369 differentially expressed genes (DEGs) (*P* ≤ 0.05, fold change ≥ 1.5) comprising 3,037 upregulated and 3,332 downregulated transcripts (Fig. 2b, Supplementary Table S3). GO analysis (Fig. 2c) revealed these DEGs were significantly enriched in transcription regulation and RNA processing (biological processes), nuclear/cytoplasmic compartments and ribosomal structures (cellular components), and protein/RNA binding activities (molecular functions). Pathway analysis (Fig. 2d) further implicated these transcriptional alterations in critical processes including cellular senescence, Hippo/FoxO signaling pathways, and neurodegenerative disease pathways, providing systematic insights into how Al exposure disrupts neuronal gene networks at multiple regulatory levels.

### Integrated analysis of m6A-Seq and RNA-Seq data of Al-induced neurotoxic effects

**Fig. 3:**
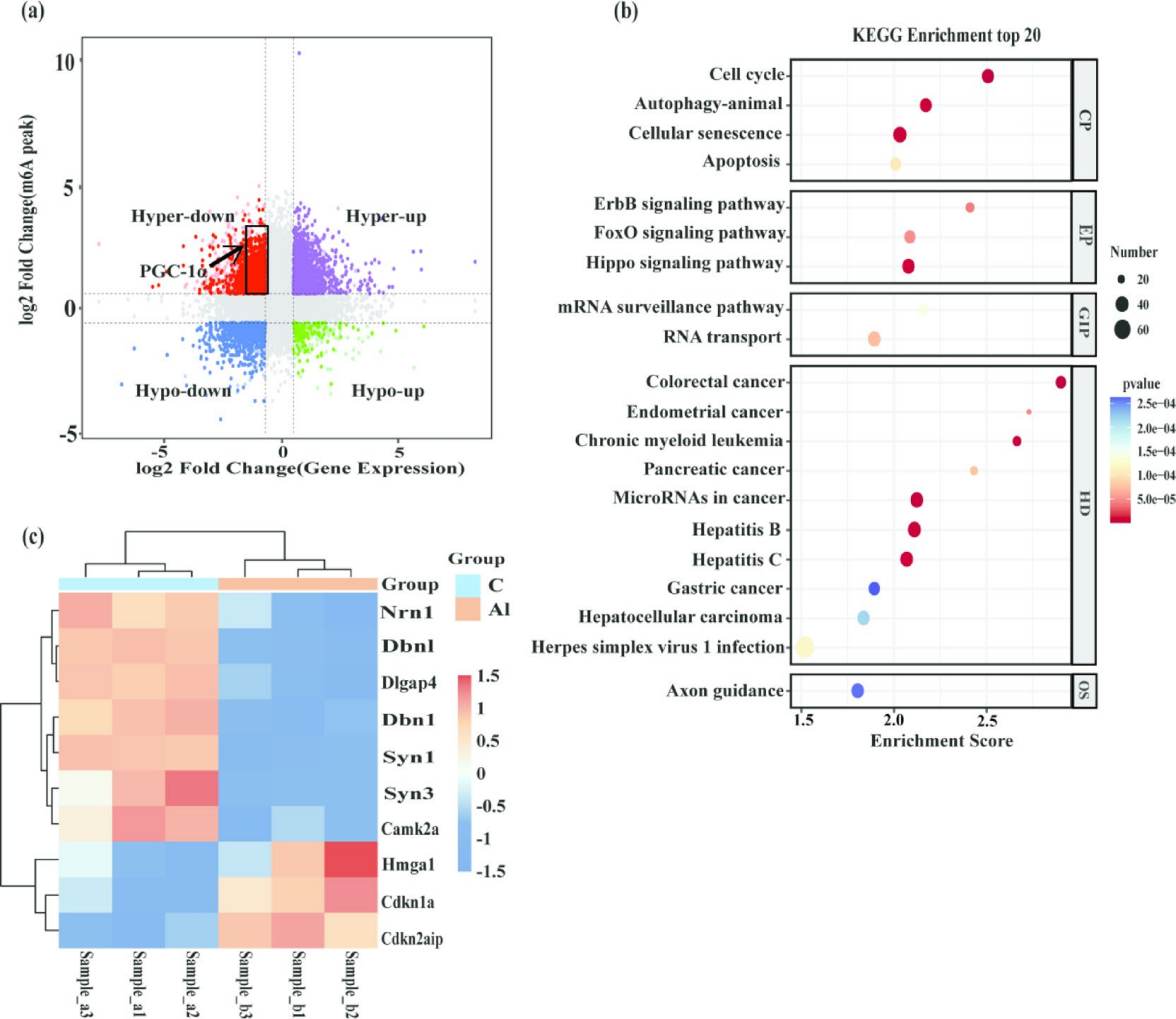
Integrated analysis of m6A methylation and gene expression. (**a**) Four-quadrant plot showing genes categorized by changes in m6A levels and mRNA expression: hyper-up, hyper-down, hypo-up, hypo-down. (**b**) KEGG enrichment of co-regulated genes indicating pathways involved in cellular senescence and neurodegeneration. (**c**) Heatmap of differentially expressed genes based on RNA-seq data following aluminum exposure. The heatmap illustrates the expression patterns of representative synaptic function–related genes and senescence-associated genes between control (C) and aluminum-treated (Al) groups. Each column represents an independent biological replicate (*n* = 3 per group). Gene expression values were Z-score normalized across samples and visualized using a color scale ranging from − 1.5 (low expression, blue) to 1.5 (high expression, red).

To systematically explore the impact of m6A modification on transcript levels, genes were categorized into four types based on combined changes in methylation and mRNA expression: hypermethylated-upregulated, hypermethylated-downregulated, hypomethylated-upregulated, and hypomethylated-downregulated. The respective numbers of genes in each category were 1,516, 1,419, 124, and 834. Pathway enrichment of these co-regulated genes highlighted cellular senescence and neurodegeneration-related signaling, with PGC-1α identified as a hypermethylated and downregulated key target. After multiple-testing correction using the Benjamini–Hochberg method (FDR < 0.05), numerous differential m6A peaks remained significant. Importantly, the m6A peaks associated with PPARGC1A (PGC-1α) also remained highly significant after FDR correction. This type contains 1516, 1419, 124 and 834 genes, respectively (Fig. 3a, Supplementary Table S4). Joint analysis identified common changing genes for KEGG pathway analysis. According to Fig. 3b, m6A modification was enriched in cellular senescence pathway. RNA-seq–based transcriptome analysis demonstrated altered expression patterns of senescence-associated genes (Cdkn2aip, Cdkn1a, and Hmga1) and synaptic function–related genes (Camk2a, Dlgap4, and Synapsins) following aluminum exposure. Heatmap visualization revealed a coordinated downregulation of synaptic genes accompanied by upregulation of senescence-related genes (Fig. 3c).Notably, PGC-1α exhibited hypermethylation with concomitant RNA downregulation (Fig. 3a), suggesting that m6A modification may suppress this mitochondrial biogenesis regulator, suggesting that m6A modification may suppress this mitochondrial biogenesis regulator, and may be associated with alterations in mitochondrial homeostasis, thereby potentially linking cellular senescence and synaptic changes through an m6A–PGC-1α–mitochondria axis.

### Effect of Al on senescence in mouse hippocampal neurons

**Fig. 4:**
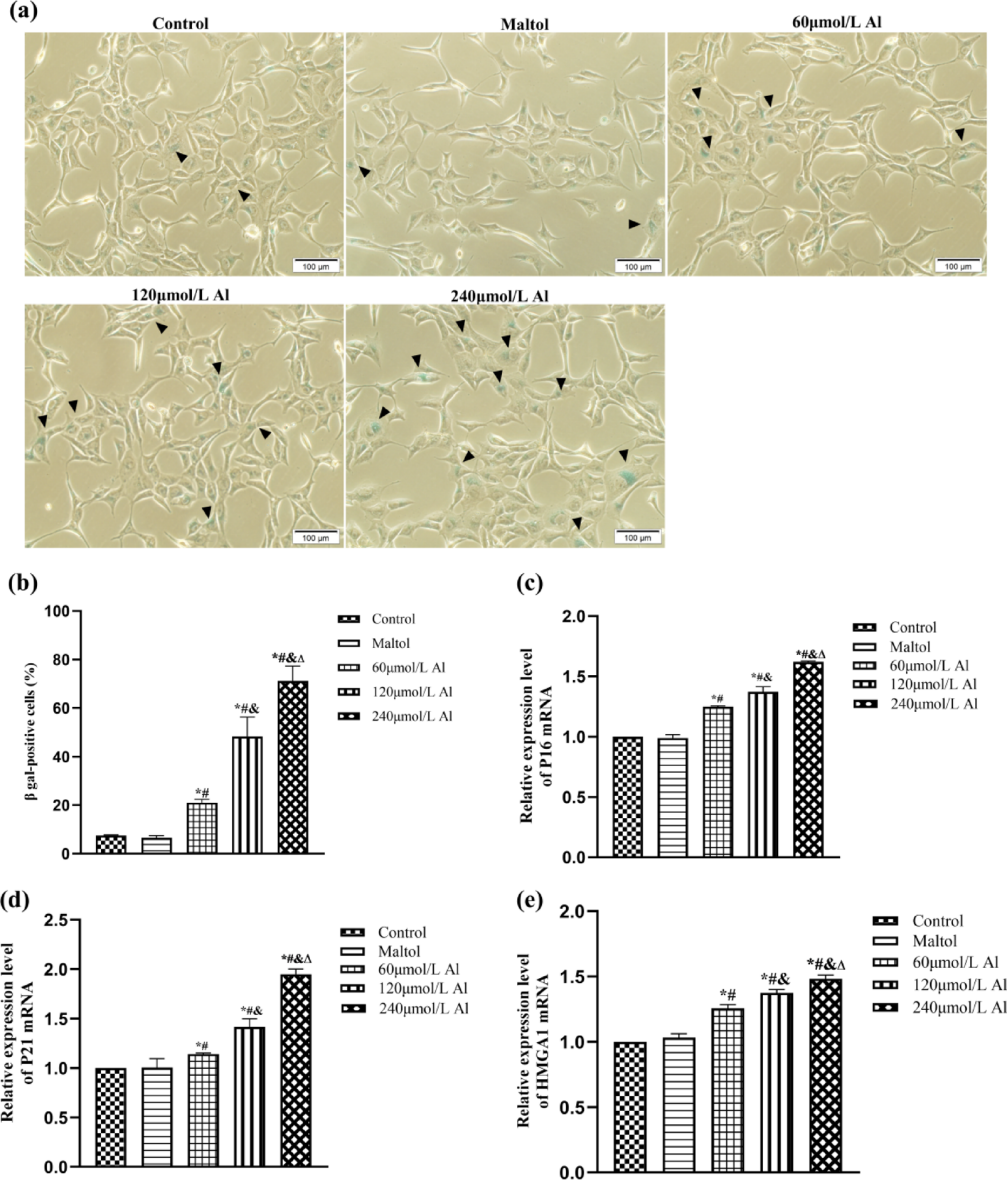
Aluminum promotes neuronal senescence in a concentration-dependent manner. (**a**) Representative SA-β-gal staining images showing senescent HT22 cells after Al treatment. (**b**) Quantification of SA-β-gal-positive cells. (**c**–**e**) mRNA expression levels of senescence markers p16, p21, and HMGA1 measured by qRT-PCR.Data are expressed as mean ± SD (*n* = 3). St atistical analysis was performed using one-way ANOVA followed by LSD post-hoc test. *: *P* < 0.05 compared with the Control group; #: *P* < 0.05 compared with the Maltol group; &: *P* < 0.05 compared with the 60 µmol/L Al group; ∆: *P* < 0.05 compared with the 120 µmol/L Al group.

Subsequently, we validated the results obtained from the inter-omics joint analysis. The experimental results showed that the β-glycosidase staining of the hippocampal neurons was positive, displaying characteristic blue-green staining around the nucleus (Fig. 4a). As shown in Fig. 4b, compared with the control group, the proportion of SA-β-gal-positive cells in Al groups exhibited a dose- dependent increase, demonstrating progressive neuronal senescence induction. Correspondingly, quantitative analysis revealed significant upregulation of senescence- associated genes in Al- treated neurons (Fig. 4c–e). The mRNA expression levels of P16 (F = 381.227, *P* < 0.05), P21 (F = 133.266, *P* < 0.05), and HMGA1 (F = 212.879, *P* < 0.05) all showed dose-responsive elevation. Specifically, compared to the control baseline (1.00 ± 0.00):P16 expression increased to 1.24 ± 0.02 (60 µmol/L Al), 1.37 ± 0.01 (120 µmol/L Al), and 1.61 ± 0.01 (240 µmol/L Al); P21 levels rose to 1.14 ± 0.01, 1.41 ± 0.08, and 1.94 ± 0.05; HMGA1 expression reached 1.25 ± 0.03, 1.37 ± 0.02, and 1.48 ± 0.02 (all *P* < 0.05), confirming molecular-level senescence activation.

### Effects of Al on FTO and mitochondrial biogenesis in mouse hippocampal neurons

**Fig. 5:**
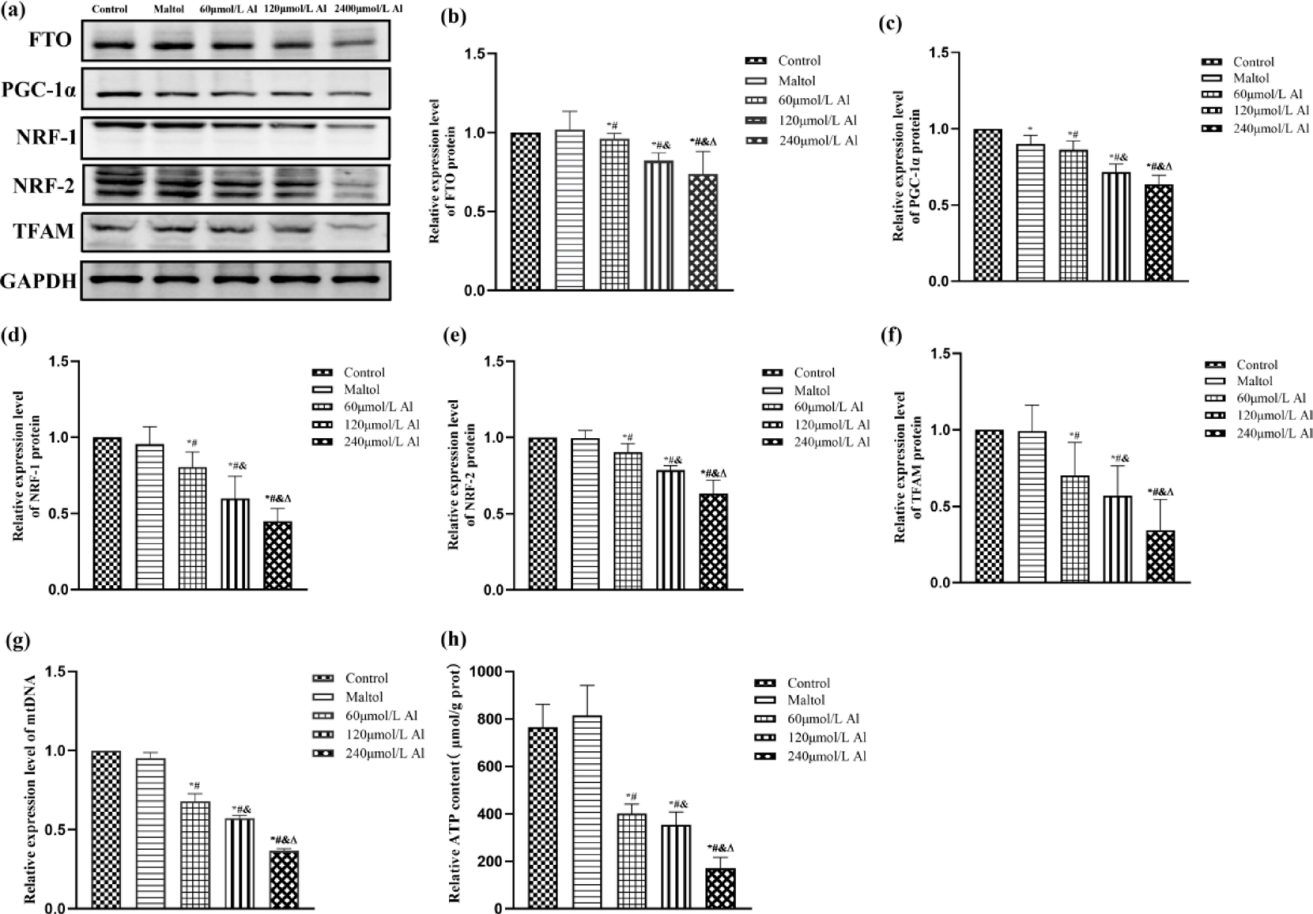
Effects of aluminum on FTO expression and mitochondrial biogenesis in HT22 cells. (**a**) Western blot images depicting the expression of FTO, PGC- 1α, NRF- 1, NRF- 2, and TFAM proteins in HT22 cells, GAPDH was used for normalization. Full-length and uncropped blots with membrane edges are provided in Supplementary Figure S1. (**b**–**f**) Statistical analysis of the relative expression levels of FTO, PGC − 1α, NRF − 1, NRF − 2, and TFAM proteins in HT22 cells. (**g**–**h**) Statistical data regarding the content of mtDNA and ATP. Control groups were regarded as 100%; data show relative quantities compared with controls. Data are expressed as mean ± SD (*n* = 3).Statistical analysis was performed using one-way ANOVA followed by LSD post-hoc test. *: *P* < 0.05 compared with the Control group; #: *P* < 0.05 compared with the Maltol group; &: *P* < 0.05 compared with the 60 µmol/L Al group; ∆: *P* < 0.05 compared with the 120 µmol/L Al group.

As systematically demonstrated in Fig. 5a, b, FTO protein expression exhibited significant dose-dependent reductions following Al exposure (120µmol/L: 0.82 ± 0.04, 240µmol/L: 0.73 ± 0.14 vs. control 1.00 ± 0.00; F = 5.926, *P* < 0.05). These results establish that Al: (1) Concentration-dependently suppresses FTO demethylase (≥ 18% reduction at neurotoxicologically relevant doses); (2) Compromises m6A erasure capacity; and (3) Drives pathological RNA methylation accumulation, collectively disrupting the FTO- mediated epitranscriptomic axis in neuronal dysfunction.

Al exposure was associated with dose-dependent alterations in mitochondrial biogenesis-related markers in HT22 cells, characterized by reduced mitochondrial biogenesis markers: (1) PGC-1α protein levels (Fig. 5a, c) exhibited progressive Al-dose suppression (F = 24.109, *P* < 0.05), decreasing to 0.86 ± 0.05 (60µmol/L), 0.71 ± 0.05 (120µmol/L), and 0.63 ± 0.06 (240µmol/L) relative to control (1.00 ± 0.00, all *P* < 0.05); (2) Mitochondrial transcription factors (Fig. 5a, d–f) followed analogous patterns- NRF-1 (F = 20.923) declined to 0.80 ± 0.09, 0.59 ± 0.14, and 0.44 ± 0.08; NRF-2 (F = 25.223) reduced to 0.90 ± 0.05, 0.78 ± 0.02, and 0.63 ± 0.08; TFAM (F = 7.767) decreased to 0.99 ± 0.17, 0.57 ± 0.19, and 0.34 ± 0.20 (all *P* < 0.05 vs. control). These coordinated changes suggest that Al may impair mitochondrial biogenesis by downregulating master regulators (PGC-1α) and their downstream transcriptional network (NRF-1/2, TFAM) by simultaneously downregulating master regulators (PGC-1α) and their downstream transcriptional network (NRF-1/2, TFAM).

Consistent with these molecular alterations, mtDNA content and ATP levels (Fig. 5g, h) were reduced in a dose-dependent manner, where mtDNA content (F = 262.070) dropped dose-dependently to 0.67 ± 0.04 (60µmol/L), 0.57 ± 0.01 (120µmol/L), and 0.36 ± 0.01 (240µmol/L) (all *P* < 0.05 vs. control 1.00 ± 0.00), while ATP levels (F = 30.742) showed severe depletion to 52% (60µmol/L: ~423.78µmol/g prot), 46% (120µmol/L: ~374.89µmol/g prot), and 22% (240µmol/L: ~179.29µmol/g prot) of control baseline (814.97 ± 96.18 µmol/g prot, all *P* < 0.05). These data suggest a potential link between Al-induced FTO suppression and alterations in PGC-1α signaling, mitochondrial biogenesis–related pathways, and cellular energetics.

### The impact of FTO overexpression on Al- induced senescence in mouse hippocampal neurons

**Fig. 6:**
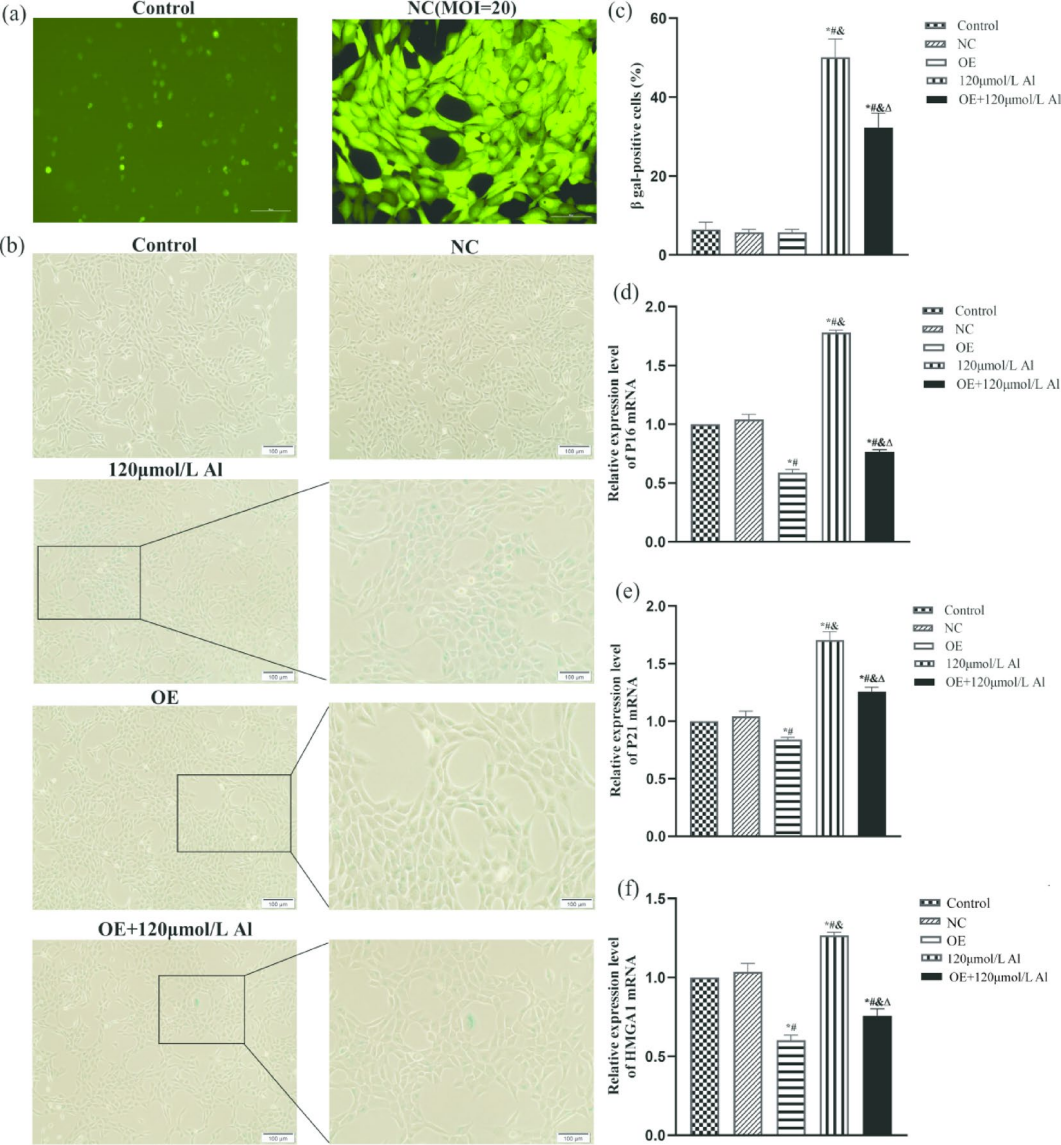
FTO overexpression attenuates Al-induced neuronal senescence. (**a**) Fluorescence images confirming FTO overexpression by lentiviral transduction. (**b**) Representative SA-β-gal staining for control, OE-FTO, Al, and OE-FTO + Al groups. (**c**) Quantification of SA-β-gal-positive cells. (**d**–**f**) Relative mRNA expression of p16, p21, and HMGA1.Control groups were regarded as 100%; data show relative quantities compared with controls. Data are expressed as mean ± SD (*n* = 3). Statistical analysis was performed using one-way ANOVA followed by LSD post-hoc test. *: *P* < 0.05 compared with the Control group; #: *P* < 0.05 compared with the NC group; &: *P* < 0.05 compared with the OE group; ∆: *P* < 0.05 compared with the 120 µmol/L Al group.

As shown in Fig. 6a, HT22 cells were transfected with a lentivirus overexpressing the FTO gene to investigate whether FTO could counteract Al- induced senescence in HT22 cells. Figure 6b, c demonstrated that, compared to the Al- treated group, the FTO-overexpressing(OE) + Al group exhibited a significant decrease in senescence- positive cells. Further analysis (Fig. 6d–f) revealed marked alterations in mRNA expression levels of senescence-related markers: P16 (*F* = 924.647, *P* < 0.05), P21 (*F* = 181.007, *P* < 0.05), and HMGA1 (*F* = 158.035, *P* < 0.055). Relative to the control group (1.00 ± 0.00), Al exposure upregulated P16 (1.78 ± 0.02, *P* < 0.05), P21 (1.70 ± 0.07, *P* < 0.05), and HMGA1 (1.26 ± 0.02, *P* < 0.05). Co-treatment with FTO and 120 µmol/L Al reversed these effects, reducing mRNA levels to 1.01 ± 0.02 (*P* < 0.05), 0.47 ± 0.03 (*P* < 0.05), and 0.50 ± 0.03 (*P* < 0.05) for P16, P21, and HMGA1, respectively, demonstrating FTO’s protective effect against Al-induced senescence.

### The impact of FTO overexpression on Al- induced damage of mitochondrial biogenesis in mouse hippocampal neurons

**Fig. 7:**
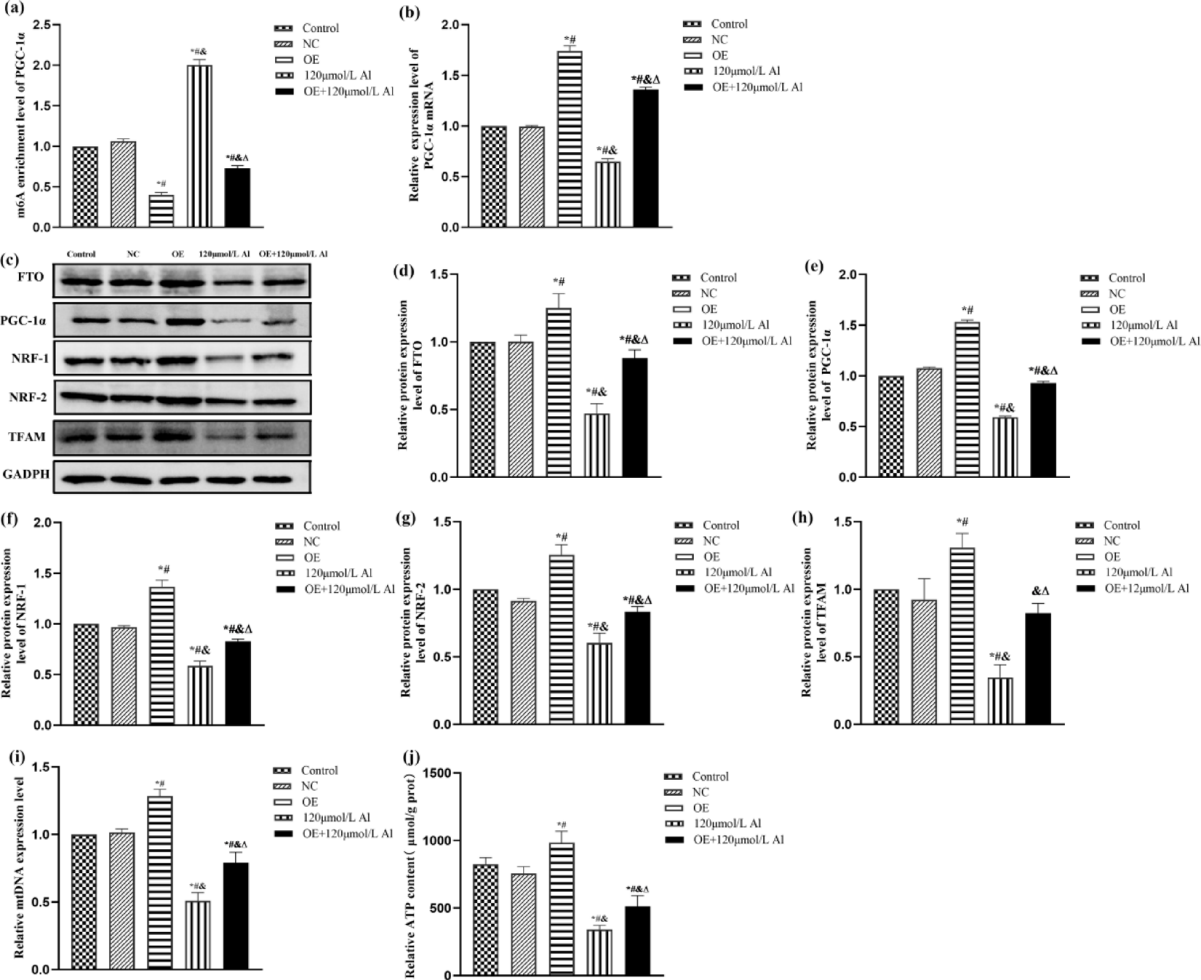
The Impact of FTO Overexpression on Al- Induced damage of Mitochondrial Biogenesis in HT22 Cells. (**a**) The effect of FTO Overexpression on the level of PGC-1α RNA m6A methylation in HT22 cells. (**b**) Relative mRNA expression levels of PGC-1α in HT22 cells under different treatments. (**c**) Western blot images showing the expression of FTO, PGC − 1α, NRF − 1, NRF − 2, and TFAM proteins in HT22 cells, GAPDH was used for normalization. Full-length and uncropped blots with membrane edges are provided in Supplementary Figure S2. (**d**–**h**) Statistical results of the relative expression levels of FTO, PGC − 1α, NRF − 1, NRF − 2, and TFAM proteins in HT22 cells, the control group was set as 100%, and the data are presented as relative quantities compared to the control. (**i**–**j**) Statistical data regarding the content of mtDNAand ATP. Control groups were regarded as 100%; data show relative quantities compared with controls.Data are expressed as mean ± SD (*n* = 3).Statistical analysis was performed using one-way ANOVA followed by LSD post-hoc test. *: *P* < 0.05 compared with the Control group; #: *P* < 0.05 compared with the NC group; &: *P* < 0.05 compared with the OE group; ∆: *P* < 0.05 compared with the 120 µmol/L Al group.

FTO-mediated regulation of mitochondrial biogenesis was further examined by integrating epitranscriptomic, transcriptional, and functional analyses (Fig. 7). (1) MeRIP-seq analysis revealed significant alterations in the m6A methylation level of PGC-1α mRNA. Compared with the control group, FTO overexpression markedly reduced m6A methylation of PGC-1α mRNA (0.40 ± 0.03, *P* < 0.05), whereas Al exposure significantly increased its m6A methylation level (2.00 ± 0.06, *P* < 0.05). In the FTO-OE + Al group, the m6A level was partially restored to an intermediate level (1.27 ± 0.03, *P* < 0.05) (Fig. 7a). Consistent with this inverse epitranscriptomic pattern, PGC-1α mRNA expression exhibited opposite changes among the treatment groups (Fig. 7b; F = 645.419, *P* < 0.05). Relative to the control group (1.00 ± 0.00), FTO-OE significantly increased PGC-1α mRNA levels (1.74 ± 0.05), whereas Al exposure markedly suppressed its expression (0.64 ± 0.02). In the FTO-OE + Al group, PGC-1α mRNA expression was partially restored (0.71 ± 0.02). (2) Protein expression of mitochondrial biogenesis regulators.Significant intergroup differences were observed in FTO protein expression (Fig. 7c, d; F = 53.619, *P* < 0.05). FTO-OE increased protein levels to 1.24 ± 0.10 compared with the control (1.00 ± 0.00, *P* < 0.05), whereas Al exposure reduced FTO expression to 0.47 ± 0.07 (*P* < 0.05). In the FTO-OE + 120 µmol/L Al group, FTO levels were partially restored to 0.88 ± 0.06 (*P* < 0.05).Consistent with the transcriptional changes, PGC-1α protein expression (Fig. 7c, e; F = 2112.044, *P* < 0.05) was significantly upregulated by FTO-OE (1.53 ± 0.01) but suppressed by Al treatment (0.59 ± 0.01). In the FTO-OE + Al group, the protein level was partially rescued (0.93 ± 0.02). Similarly, downstream mitochondrial biogenesis regulators, including NRF-1 (F = 173.141), NRF-2 (F = 67.323), and TFAM (F = 58.271), exhibited comparable trends, with FTO overexpression reversing the Al-induced suppression (all *P* < 0.05).

(3) Functional validation of mitochondrial biogenesis (Fig. 7i–j).Functional assays further supported these molecular findings. FTO-OE significantly increased mtDNA content (1.28 ± 0.05 vs. control 1.00 ± 0.00, *P* < 0.05) and ATP production (+ 19% relative to the control baseline of 824.10 ± 49.91 µmol/g prot). Moreover, in the FTO-OE + Al group, FTO overexpression mitigated the Al-induced reductions in mitochondrial function, as reflected by increased mtDNA content (0.77 ± 0.04 vs. Al 0.59 ± 0.06) and ATP levels (+ 49% vs. Al 341.38 ± 79.62 µmol/g prot) (both *P* < 0.05).

Collectively, these results indicate that FTO plays an important regulatory role in neuronal metabolism, potentially through modulation of the m6A–PGC-1α axis, thereby alleviating Al-induced impairment of mitochondrial biogenesis–related pathways.

## Discussion

In the present study, we showed that Al exposure is associated with neuronal senescence involving a potential epitranscriptomic mechanism related to the FTO–m6A–PGC-1α axis. Our findings suggest that downregulation of the m6A demethylase FTO is associated with impaired mitochondrial biogenesis, providing a potential epitranscriptomic perspective on aluminum-related neurotoxicity^24^. While Al neurotoxicity has been traditionally linked to oxidative stress and DNA damage^25–27^, our results suggest that the dysregulation of m6A modification represents a more sustained regulatory shift. This mechanism may partly explain why pathological changes such as amyloid-β deposition remain difficult to reverse even after antioxidant intervention^28^. Furthermore, while pro-inflammatory responses are known to be partially alleviated by therapeutics^29^, the persistence of neuronal damage after the cessation of Al exposure^30^ may be associated with alterations in the FTO/PGC-1α signaling axis observed in our in vitro model.

In this context, N6-methyladenosine (m6A), a dynamic and reversible epitranscriptomic modification, has emerged as a key regulator of neural development, synaptic plasticity, stress adaptation, and aging^31^. Dysregulation of m6A modification has been reported to affect mRNA stability, translation efficiency, and cellular stress responses, thereby reshaping neuronal functional states^32,33^. Importantly, m6A-mediated post-transcriptional regulation represents a potential mechanism contributing to the long-lasting and progressive nature of Al-induced neurotoxicity. However, whether Al exposure disrupts the m6A regulatory machinery—particularly through m6A demethylases such as FTO—and thereby impairs mitochondrial homeostasis remains largely unexplored. This knowledge gap provided the rationale for the present study.

In the present study, MeRIP-seq revealed significant enrichment of m6A modifications within the coding sequence (CDS) of mRNAs in hippocampal neurons following Al exposure.This distribution pattern is consistent with previous reports suggesting that m6A may regulate translation efficiency and transcript stability^24,25^. However, due to the limited resolution of MeRIP-seq and the lack of site-specific validation, it remains unclear whether these modifications directly affect mRNA stability or decay. In addition, potential m6A sites in the 3′UTR, which are often associated with mRNA degradation, could not be reliably resolved. Therefore, our findings support an association between m6A modification and transcript regulation rather than a direct functional link. Motif analysis further confirmed that m6A sites were enriched at the conserved RGAAR sequence, indicating stress-dependent regulatory specificity^26,27^. We identified a total of 8,936 differentially DMPs, with over 75% being upregulated, suggesting that Al, as an environmental toxin, can systemically perturb the m6A methylation landscape. Functional enrichment analysis revealed that these differentially methylated genes are significantly involved in pathways such as “transcriptional regulation,” “cell cycle,” “cellular senescence,” and “neurodegenerative diseases,” indicating that m6A may act as a regulatory factor to reprogram cell fate under stress conditions^28,29^.

Integration with RNA-seq data revealed substantial transcriptional alterations following Al exposure, with 3,037 upregulated and 3,332 downregulated genes (DEGs). These DEGs were enriched in pathways related to mRNA processing, ribosome function, FoxO, Hippo signaling, and cellular senescence^30,31^, indicating widespread remodeling of transcriptional networks related to cellular homeostasis and lifespan regulation, potentially contributing to mitochondrial impairment and activation of senescence.

Cellular senescence is a critical early event in neurodegeneration, triggered by multiple stressors such as oxidative stress and energy imbalance. It is characterized by increased SA-β-Gal activity, elevated p16 and p21 expression, and the development of the senescence- associated secretory phenotype (SASP)^32–34^. Studies have confirmed that both neurons and glial cells can undergo senescence, exhibiting typical senescent phenotypes in the brains of AD and PD patients^35–38^. In our study, Al led to a dose-dependent increase in SA-β-Gal-positive hippocampal neurons, as well as upregulation of p16, p21, and HMGA1 expression, indicating that Al activates neuronal senescence. Further analyses suggest that mitochondrial impairment—particularly reduced mitochondrial biogenesis—may act as a key mediator of Al-induced neuronal senescence. Mitochondrial biogenesis impairment is associated with reduced ATP production and oxidative stress, thereby potentially accelerating senescence^39,40^. Through integrative analysis of MeRIP-seq and RNA-seq data, we identified 2,723 co-regulated genes that showed both altered m6A methylation and differential expression. Among them, PGC-1α exhibited a high-methylation and low-expression pattern, suggesting a potential association between m6A modification and transcript regulation^23,41^. In addition, we observed that PGC-1α mRNA levels were reduced in parallel with protein expression (Fig. 7b), suggesting coordinated regulation at the transcriptional and/or post-transcriptional level. However, we did not assess mRNA stability or translation efficiency, and therefore cannot determine the precise regulatory mechanism. In addition, no quantitative correlation analysis between m6A enrichment and transcript abundance was performed. Moreover, decreased expression of synaptic proteins such as CaMK2, PSD-95, and Synapsin further indicated disruption of synaptic homeostasis by Al.

Importantly, we uncovered a central role for the FTO–PGC-1α–mitochondrial axis in Al- induced neuronal senescence. FTO, an m6A demethylase, plays a key role in neuronal energy metabolism and stress responses, and its downregulation has been reported in various neural injury models^42,43^. PGC-1α is a master transcriptional regulator of mitochondrial biogenesis; its dysfunction directly compromises cellular energy production and antioxidant capacity^44^. In our study, Al exposure dose-dependently reduced FTO expression, which led to increased m6A methylation on PGC-1α mRNA and reduced PGC-1α protein levels. Although the downstream consequences of FTO suppression were characterized, the upstream mechanisms by which aluminum exposure reduces FTO expression remain unclear. In the present study, we primarily assessed FTO at the protein level, and parallel measurement of FTO mRNA was not systematically performed. Therefore, it cannot be determined whether the observed downregulation is driven by transcriptional repression or post-transcriptional mechanisms, such as altered mRNA stability or protein degradation, which has been reported to regulate FTO protein abundance in certain cellular contexts^45,46^.

Because aluminum exposure induces oxidative and mitochondrial stress responses, these signals may contribute to reduced FTO expression, consistent with previous reports showing downregulation of FTO under oxidative stress conditions in neuronal and ischemic injury models^47,48^. Consistently, reduced FTO expression was associated with decreased levels of downstream mitochondrial regulators (NRF-1, NRF-2, TFAM), lower mtDNA copy number, and reduced ATP levels. Decreased ATP production and mtDNA copy number were observed following aluminum exposure, reflecting impaired mitochondrial biogenesis rather than direct mitochondrial function. Thus, our results primarily indicate impaired mitochondrial biogenesis rather than direct mitochondrial dysfunction. Future studies using OCR and mitochondrial membrane potential assays are needed to comprehensively assess mitochondrial function. Collectively, our results indicate that disruption of the FTO–m6A–PGC-1α axis is associated with mitochondrial biogenesis impairment and neuronal senescence induced by aluminum exposure. We further validated the reversibility of this pathway through FTO overexpression experiments. Upregulation of FTO reduced m6A methylation on PGC-1α mRNA, alleviating m6A-associated repression^49^. This was associated with increased PGC-1α protein levels, a critical transcriptional coactivator for mitochondrial biogenesis^22^. Reactivated PGC-1α promoted transcription of mitochondrial genes, partially restored mtDNA replication and ATP production, and improved redox balance^22^. FTO overexpression restored mitochondrial integrity and energy homeostasis, counteracting stress-induced activation of p16^INK4a, p21^CIP1, and HMGA1-mediated SAHF formation. SA-β-Gal activity decreased, confirming neuronal functional rescue^21^. These findings support that FTO-mediated m6A demethylation provides a regulatory mechanism linking RNA methylation dynamics to mitochondrial biogenesis and senescence signaling.

While this study identifies the FTO/m6A/PGC-1α axis as a key mediator of aluminum (Al)-induced neurotoxicity, several mechanistic questions remain unresolved. The upstream processes by which Al exposure suppresses FTO expression in hippocampal neurons require further investigation. In addition, we did not systematically assess the expression or activity of m6A “writers” (e.g., METTL3, METTL14, and WTAP), and coordinated regulation of m6A methylation machinery therefore cannot be excluded. Whether m6A-dependent repression of PGC-1α results from altered mRNA stability or translational efficiency also warrants clarification. Furthermore, while we observed impaired mitochondrial biogenesis and reduced ATP production, more direct functional evidence—such as changes in mitochondrial membrane potential, reactive oxygen species (ROS) levels, and real-time respiratory chain activity (e.g., Seahorse extracellular flux analysis)—was not comprehensively evaluated in this study. Moreover, given the pleiotropic nature of FTO, PGC-1α-independent pathways may contribute to mitochondrial protection, which should be addressed in future studies.

From a translational perspective, therapeutic targeting of the FTO–m6A–PGC-1α axis must carefully balance efficacy and safety. Because FTO regulates a broad range of transcripts involved in metabolism and neuronal function, global modulation of FTO activity may lead to off-target or systemic effects. Thus, future therapeutic strategies should favor cell type–specific or pathway-focused interventions, such as selectively enhancing downstream mitochondrial regulators including PGC-1α, rather than indiscriminate manipulation of FTO itself. Such precision-oriented approaches may maximize neuroprotective benefits while minimizing adverse consequences.

Despite these insights, several experimental limitations must be acknowledged. Future studies combining transcript decay assays, ribosome profiling, and site-specific mapping of m6A residues will be required to clarify the post-transcriptional regulation of PGC-1α. Furthermore, while HT22 cells are an established model for studying neuronal oxidative stress and senescence, their use as an immortalized cell line limits the physiological relevance of our findings. This study focused on the initial discovery of the FTO/m6A mechanism in a controlled in vitro environment. However, the complexity of the intact brain cannot be fully recapitulated by cell culture systems alone. Therefore, further validation in primary neuronal cultures and in vivo animal models is essential to confirm the translational significance of the FTO–m6A–PGC-1α axis in aluminum-induced neurodegeneration.

## Conclusions

Aluminum exposure upregulates m6A methylation by inhibiting the demethylase FTO, reduces PGC-1α expression, and suppresses mitochondrial biogenesis, as evidenced by decreased NRF-1/NRF-2/TFAM expression and reduced mtDNA and ATP levels, thereby inducing senescence in mouse hippocampal neurons.

## Electronic Supplementary Material

Below is the link to the electronic supplementary material.

Supplementary Material 1

Supplementary Material 2

Supplementary Material 3

Supplementary Material 4

Supplementary Material 5

Supplementary Material 6

Supplementary Material 7

## Data Availability

The datasets generated and analyzed during the current study have been deposited in the NCBI Gene Expression Omnibus (GEO) under the accession numbers GSE304247 and GSE304248.As the datasets are currently under embargo, they are available to editors and reviewers via the following links: GSE304247: https://www.ncbi.nlm.nih.gov/geo/query/acc.cgi?acc=GSE304247&token=qbqjaqsqtlynnun. GSE304248: https://www.ncbi.nlm.nih.gov/geo/query/acc.cgi?acc=GSE304248&token=kroramqqfrshhib.

## Abbreviations

Al: Aluminum
FTO: Fat mass and obesity-associated protein
PGC-1α: Peroxisome proliferator-activated receptor gamma coactivator-1α
NRF-1: Nuclear respiratory factor 1
NRF-2: Nuclear respiratory factor 2
TFAM: Mitochondrial transcription factor A
m6A: N6-methyladenosine
mtDNA: Mitochondrial DNA
Al(mal)_3_: Aluminum maltol
WB: Western blot
RT-PCR: Realtime polymerase
