# Supplementary material for: FTO-mediated m6A demethylation regulates PGC-1α-dependent mitochondrial biogenesis to attenuate aluminum-induced neuronal senescence
Source: Sci Rep. 2026 May 7;16:21890. doi: 10.1038/s41598-026-51674-w (PMC13365551; doi:10.1038/s41598-026-51674-w)
Supplement: Supplementary file 1 — Supplementary Material 1 [file 41598_2026_51674_MOESM1_ESM.docx]

**Figures 5a**

the first time


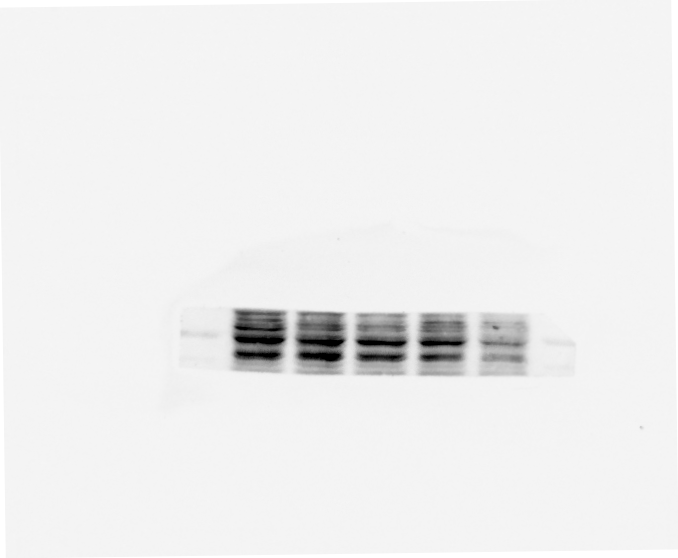


100kDa

NRF-2


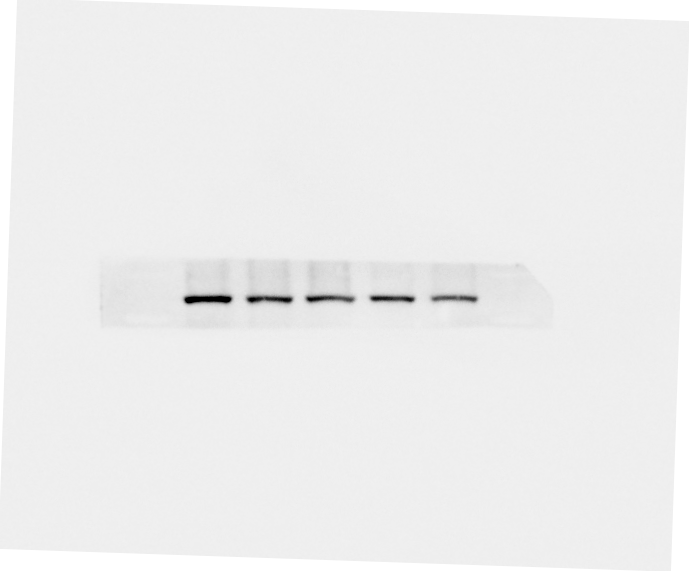


92kDa

PGC-1α


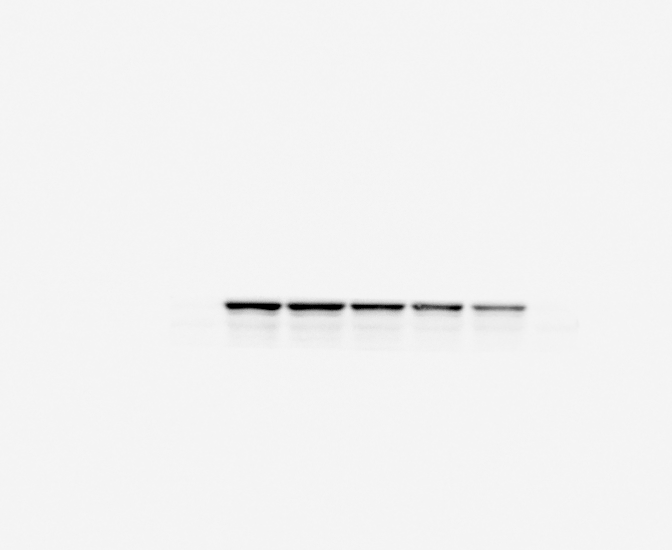


NRF-1

67kDa


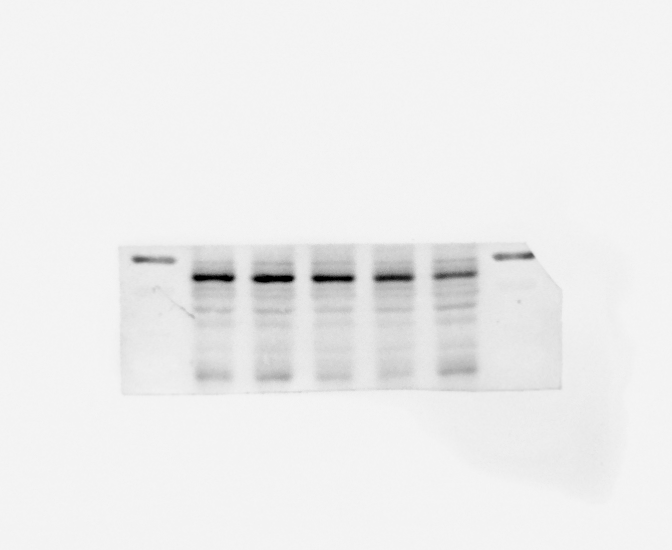


58kDa

FTO

GAPDH


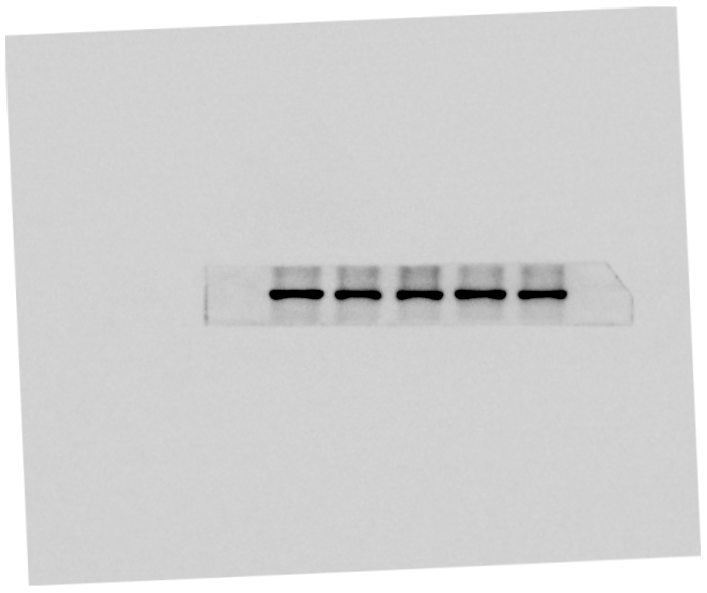


36kDa


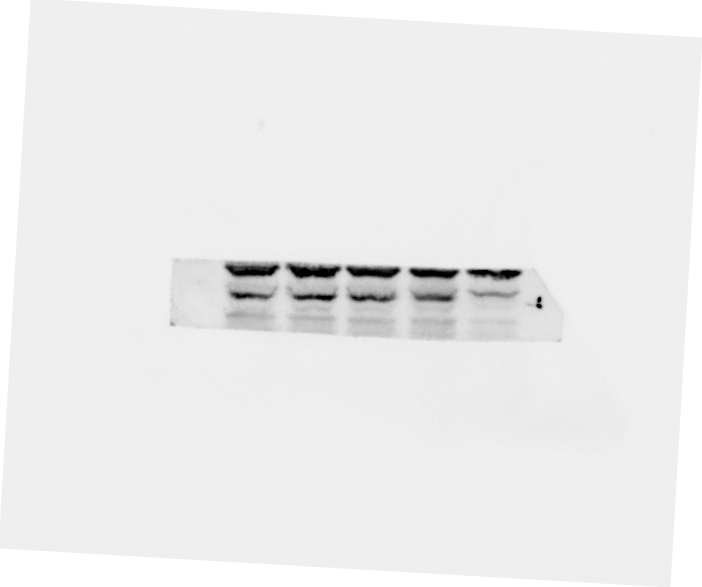


TFAM

26kDa

the second time


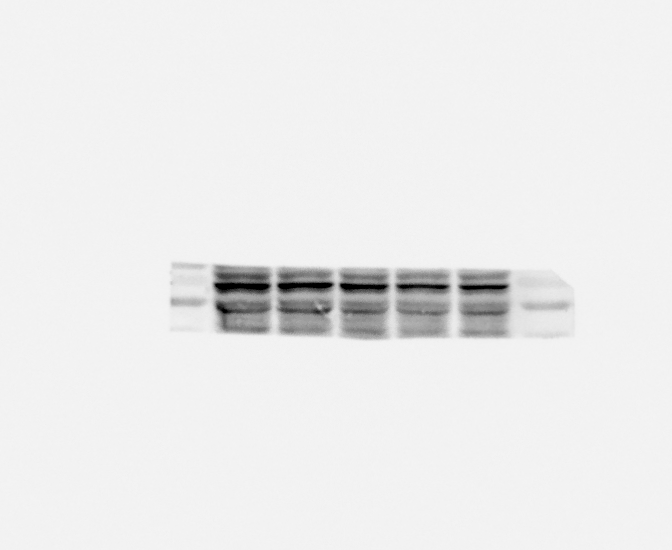


100kDa

NRF-2

PGC-1α


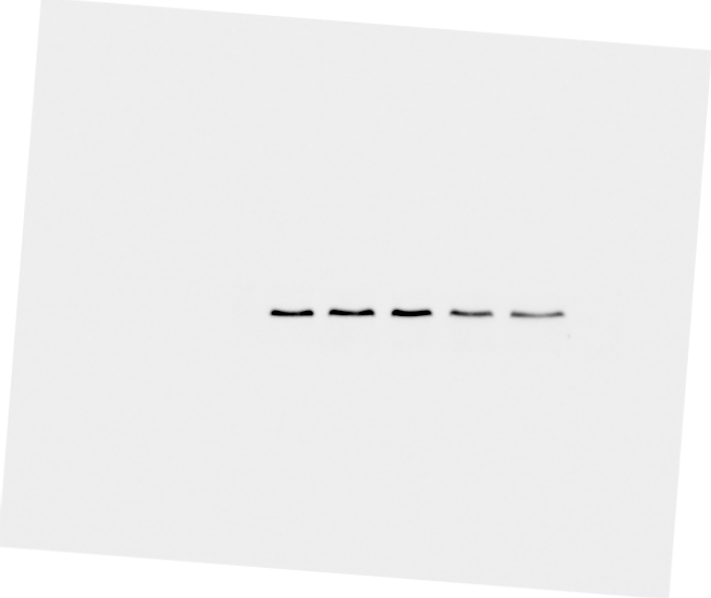


92kDa

NRF-1


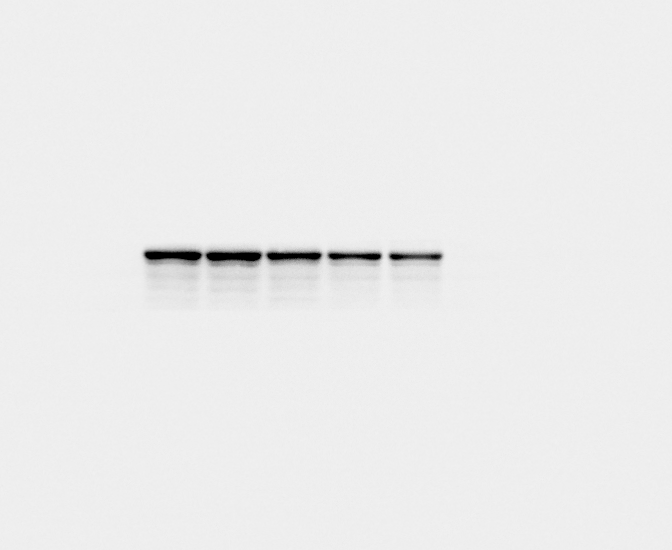


67kDa


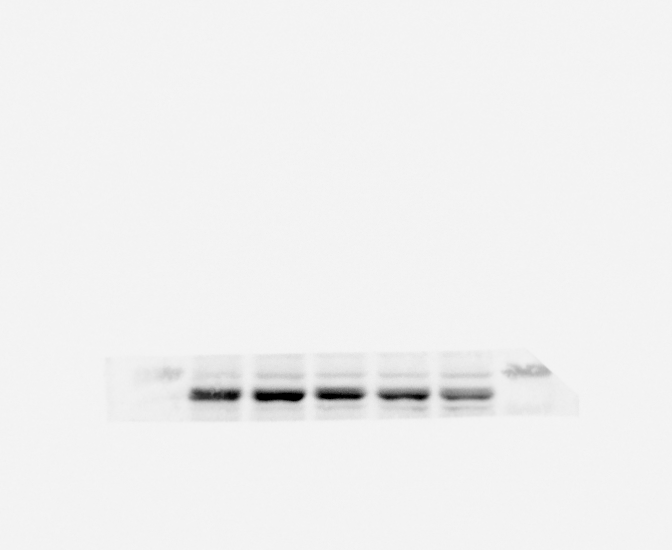


FTO

58kDa


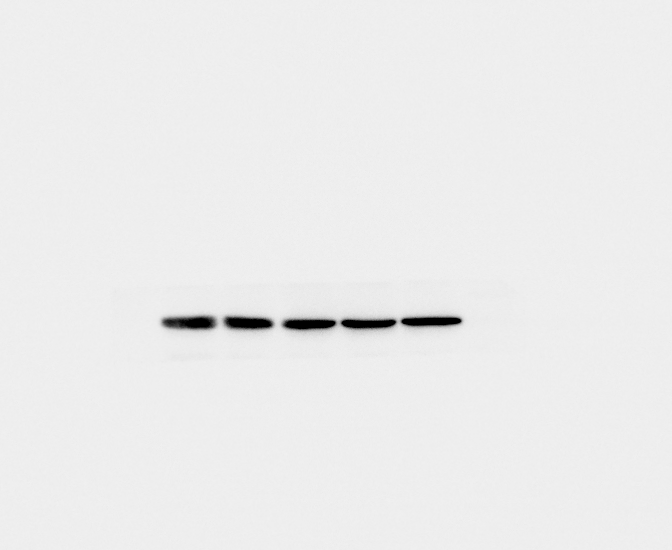


GAPDH

26kDa


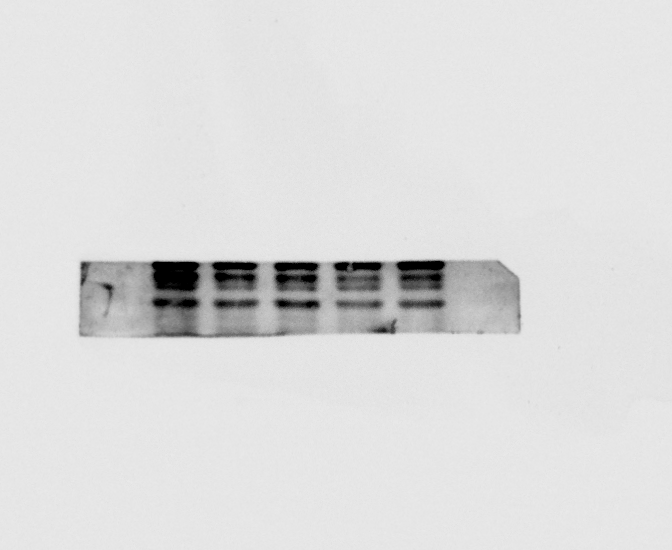


TFAM

36kDa

the third time


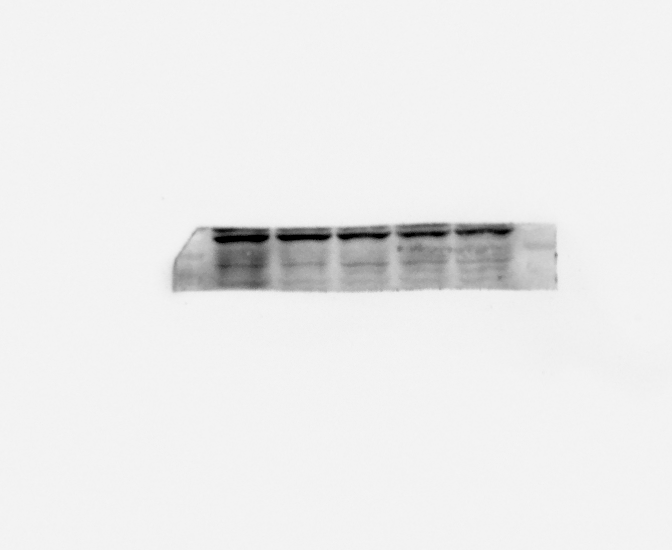


100kDa

NRF-2


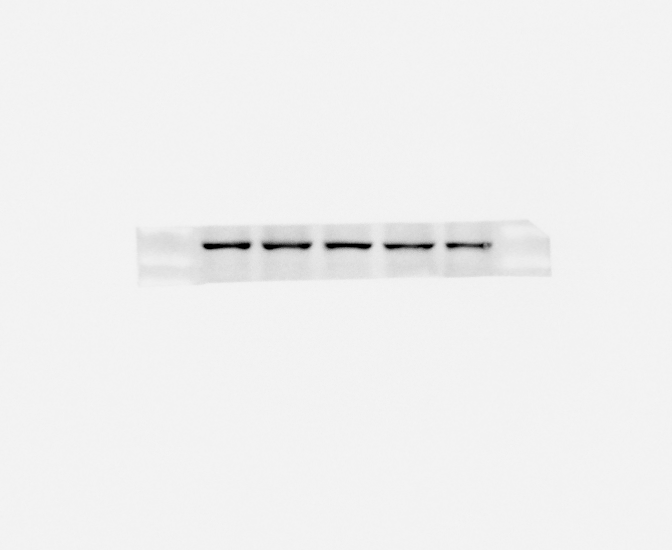


92kDa

PGC-1α


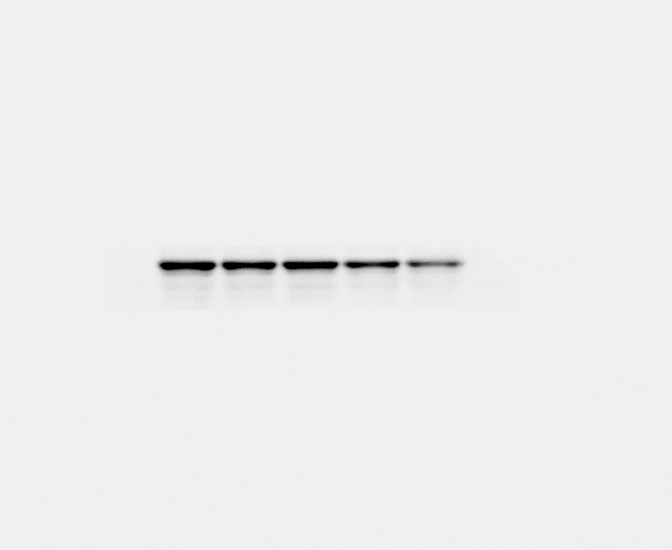


NRF-1

67kDa


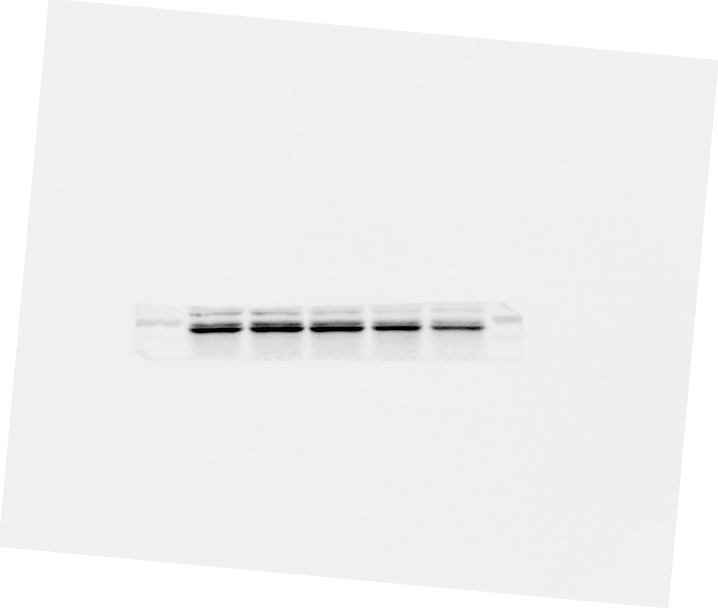


58kDa

FTO

GAPDH


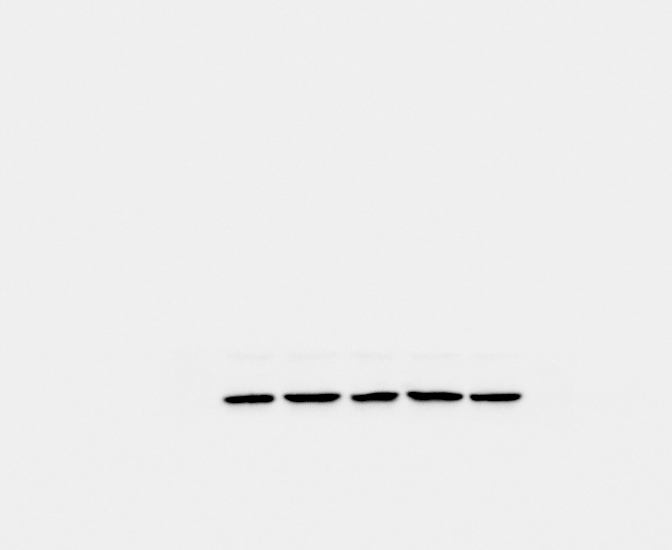


36kDa

TFAM


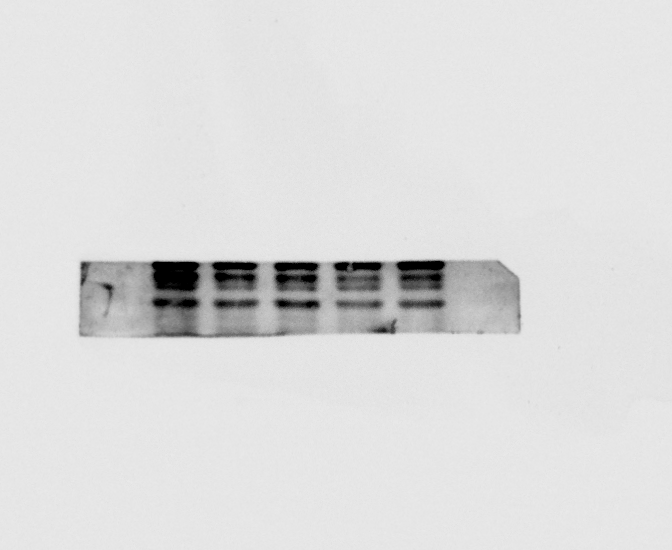


26kDa
